# Single stranded (ss)RNA-mediated antiviral response against infectious laryngotracheitis virus infection

**DOI:** 10.1186/s12866-019-1398-6

**Published:** 2019-02-08

**Authors:** Mohamed Sarjoon Abdul-Cader, Upasama De Silva Senapathi, Hanaa Ahmed-Hassan, Shayan Sharif, Mohamed Faizal Abdul-Careem

**Affiliations:** 1 Faculty of Veterinary Medicine, Health Research Innovation Center 2C53, University of Calgary, 3330 Hospital Drive NW, Calgary, AB T2N 4N1 Canada; 2 Department of Pathobiology, University of Guelph, Guelph, ON N1G 2W1 Canada

**Keywords:** Resiquimod, ssRNA, Chicken, Macrophage, Infectious laryngotracheitis virus, Nitric oxide, Type 1 interferon, Interleukin 1β

## Abstract

**Background:**

Single stranded ribonucleic acid (ssRNA) binds to toll-like receptor (TLR)7 leading to recruitment of immune cells and production of pro-inflammatory cytokines, which has been shown in mammals. In chickens, synthetic ssRNA analog, resiquimod, has been shown to elicit antiviral response against infectious bursal disease virus infection. The objective of this study was to determine the innate host responses activated by the pre-hatch *in ovo* administration of resiquimod against infectious laryngotracheitis virus (ILTV) infection in chickens post-hatch.

**Results:**

First, we observed that *in ovo* treatment of resiquimod at embryo day (ED) 18 increases macrophage recruitment in respiratory and gastrointestinal tissues of chicken day 1 post-hatch in addition to interleukin (IL)-1β in lungs. Second, we observed that *in ovo* treatment of resiquimod reduces ILTV cloacal shedding at 7 days post-infection (dpi) when challenged at day 1 post-hatch coinciding with higher macrophage recruitment. In vitro, we found that resiquimod enhances production of nitric oxide (NO) and IL-1β and not type 1 interferon (IFN) activity in avian macrophages. Although, the antiviral response against ILTV is associated with the enhanced innate immune response, it is not dependent on any of the innate immune mediators observed as has been shown in vitro using avian macrophage.

**Conclusion:**

This study provides insights into the mechanisms of antiviral response mediated by resiquimod, particularly against ILTV infection in chicken.

## Background

The innate immune system, which is known to elicit broader nonspecific protective host responses against invading pathogens, is equipped with a range of immune cells. One of the major immune cells involved in the recognition and the elimination of microbes such as viruses, bacteria, and fungi, are macrophages. Macrophages recognize these pathogens, through pattern recognition receptors (PRRs) including toll-like receptors (TLRs) [1–3]. These TLRs are able to detect the presence of specific molecular patterns, known as pathogen-associated molecular patterns (PAMPs) [4–7] that are highly conserved amongst microbes.

When a virus enters a host, viral PAMP-TLR interaction activates intracellular signaling cascades [8] leading to upregulation of gene transcription initiating innate immune responses, including antiviral cytokines such as type-1 interferons (IFNs), interleukin (IL)-1β and inducible nitric oxide synthase (iNOS) [9–11]. The latter enzyme facilitates production of highly reactive and potent antiviral molecule, nitric oxide (NO), as a part of innate host defense against invading infectious agents [12, 13].

In chickens, the activation of different types of TLRs using synthetic ligands induces innate antiviral response. For example, *in ovo* treatment of CpG DNA significantly induced the recruitment of macrophages in chicken lungs [13]. This induction was associated with the inhibition of avian influenza virus replication in a NO dependent way. Similarly, in another study, the enhanced NO production in macrophages following treatment of the TLR4 ligand, lipopolysaccharide (LPS), led to an antiviral response against infectious laryngotracheitis virus (ILTV) [14]. Furthermore, *in ovo* treatment of TLR2 ligand, lipotechoic acid (LTA), reduced ILTV infection in chickens which correlated with a significant upregulation of mRNA expression of pro-inflammatory mediators such as IL-1β and iNOS [15].

Of the TLRs in birds, TLR7 is the only identified receptor that binds with viral single-stranded ribonucleic acid (ssRNA) or its synthetic analogs (such as resiquimod, imiquimod, gardiquimod and loxoribine) [11, 16]. In chickens, ssRNA can induce antibacterial effects against *Salmonella Enteritidis* [17] and antiviral effects against very virulent infectious bursal disease virus infection [18], similarly in mice, pre-treatment with resiquimod cleared bacteria involved in sepsis [19]. Recently, a study demonstrated that synthetic ssRNA upregulates mRNA of pro-inflammatory mediators including IL-1β and iNOS in chicken in vivo [20]. However, the antiviral response of TLR7 activation against ILTV infection in chicken is not known. Therefore, our objectives for this study were to determine 1) whether *in ovo* treated synthetic ssRNA, resiquimod is capable of eliciting macrophage responses post-hatch, 2) whether activation of the TLR7 pathway stimulates antiviral activity against ILTV and 3) the antiviral mechanisms involved following activation of TLR7 pathway in chicken.

## Materials and methods

### Animals

The use of specific pathogen free (SPF) eggs, chickens, and embryos in all our experimental procedures were approved by the Health Science Animal Care Committee (HSACC). The SPF eggs were purchased from the Canadian Food Inspection Agency (CFIA, Ottawa, ON, Canada) and incubated at 37.2 °C at 60% relative humidity for 18 days and then at 37.6 °C and 70% relative humidity for last 3 days of incubation [9] in digital incubators (Rcom Pro 20 and 50, Kingsuromax 20 and Rcom MARU Deluxe max, Autoelex Co., Ltd., GimHae, GyeongNam, Korea). The eggs were candled at embryo day (ED) 11 in order to select fertile eggs for the experiments. The chickens were euthanized before sampling of tissues as has been approved by the institutional animal care committees. Briefly, the chickens were euthanized using overdose of isoflurane anesthesia followed by cervical dislocation before sampling of tissues post-hatch.

### Virus and TLR ligand

The ILTV used in the studies was purchased from the American Type Culture Collection (strain N-71851, ATCC, Manassas, Virginia, United States). Initially, the virus was propagated in embryonated chicken eggs at ED 9–11 by infecting them through the chorioallantoic membrane (CAM) route and a plaque assay was performed using leghorn chicken hepatocellular carcinoma (LMH) cells (ATCC, Manassas, Virginia, United States) to determine the viral titer in the harvested allantoic fluid. The vesicular stomatitis virus (VSV) encoded with green fluorescent proteins (GFP) was kindly provided by Dr. Markus Czub, University of Calgary. The ligand for TLR7, synthetic ssRNA, resiquimod, was purchased from *Selleckchem* (Houston, TX, USA).

### Cells and cell culture

The Muquarrab Qureshi-North Carolina State University (MQ-NCSU) cell line [21], a macrophage cell line, was kindly gifted by Dr. Shayan Sharif (University of Guelph, Guelph ON, Canada). This cell line was cultured in LM-HAHN media as has been described previously [13]. Both Douglas Foster (DF)-1 [22] chicken fibroblast and LMH cell lines, purchased from American Type Culture Collection (ATCC, Manassas VA, USA), were cultured in Dulbecco’s Modified Eagle’s Medium (DMEM) supplemented with penicillin (100 units/ml), streptomycin (100 μg/ml) and 10% fetal bovine serum (FBS), in a humidified incubator at 40 °C and 5% CO_2._.

### *In ovo* treatment technique

In the poultry industry, *in ovo* injection at ED18 has become a standard route of vaccination, inducing early immune responses compared to post-hatch vaccination [23]. In agreement with this practice, we treated resiquimod *in ovo* as has been described previously [13, 23, 24]. Briefly, the shell surface of the eggs was disinfected with 70% ethanol and the egg shell was punctured through air sac at the broader end of the eggs using a 21-gauge needle. For *in ovo* treatment of compounds into the amniotic cavity, the entire length of the needle was inserted perpendicularly through the punctured hole using a sterile 23-gauge, 2½ centimeter long needle. At the end of the procedure the punctured holes in the shell of the egg were sealed with lacquer before placing the eggs in the egg incubator.

### Experimental design

#### Determination whether *in ovo* treatment of resiquimod elicits macrophage responses and NO production post-hatch

During the incubation at ED18, 100 μg resiquimod diluted in 200 μl of sterile PBS was administered at ED18 *in ovo* in 5 SPF chicken eggs, while the control group (*n* = 4) received 200 μl sterile PBS per egg. Then, the eggs were continued to incubate until they hatched. Following euthanizing at day 1 post-hatch, one lung was collected in PBS for NO assay. The NO assay was performed in lung supernatant collected after homogenization and centrifugation using Griess reagent system as described under a separate title below. The other lung, trachea, duodenum and large intestine were collected and preserved in optimum cutting temperature (OCT) compound (Tissue-Tek®, Sakura Finetek USA inc, Torrance CA, USA) at − 80 °C before being sectioned for immunostaining as described below under the title of “immunofluorescent assay”.

#### Determination whether *in ovo* treatment of resiquimod elicits an antiviral response against ILTV post-hatch

During the incubation, 100 μg resiquimod diluted in 200 μl of sterile PBS was administered *in ovo* at ED18 in 6 SPF chicken eggs, while the control group (*n* = 6) received 200 μl sterile PBS per egg. Then, the treated and control eggs were continued to incubate until they hatched. Both groups were infected with 3 × 10^4^ plaque forming units (PFU) of ILTV/chicken intra-tracheally at day 1 post-hatch and the chickens were observed for 12 days post-infection (dpi). At the end of the experiment, the birds were euthanized as described above. Oropharyngeal and cloacal swabs were collected at 4 and 7 dpi, and DNA extraction was performed to quantify ILTV genome loads as described under a separate title below.

#### Stimulation of macrophage cells with resiquimod for quantification of NO production

MQ-NCSU cells were propagated in 12-well plates (1 × 10^6^ cells per well) for 24 h and subsequently incubated at 40 °C and 5% CO_2_ with Roswell Park Memorial Institute (RPMI) media containing resiquimod at 50, 25, 10, 5, 2 or 0 μg/mL. All experiments were performed in triplicates. LPS (1 μg/mL) was used as a positive control. Culture supernatants were collected for NO assay at 24 h post-treatment.

#### Determination whether resiquimod-mediated antiviral response against ILTV is attributable to NO originated from macrophages

A selective inhibitor of iNOS, N-([3-(Aminomethyl) phenyl] methyl) ethanimidamide dihydrochloride (1400 W) (Sigma-Aldrich, St. Louis MO, USA) [25, 26] was used to block NO production. Initially, MQ-NCSU cells were cultured in 12-well plates for 24 h and stimulated with RPMI medium containing either resiquimod (10 μg/ml), 1400 W (100 μM), resiquimod (10 μg/ml) combined with 1400 W (100 μM) or RPMI growth media only as control. Each group was treated in triplicate. Meanwhile, the LMH cell was cultured (2.5 × 10^6^ cells per well) in a separate 12-well plate for 24 h. The MQ-NCSU culture supernatants were collected at 24 h post-treatment. The LMH cell monolayer was washed with HBSS once and 250 μl of MQ-NCSU culture supernatant collected was transferred directly to an LMH cell monolayer and immediately infected with ILTV directly into the transferred supernatant at a multiplicity of infection (MOI) of 0.0012 (300 PFUs/well). After infection, the LMH cells were incubated for 2 h in a humidified incubator at 37 °C before adding DMEM culture media. As negative controls for the macrophage culture supernatants, we used RPMI media. At 5 dpi, the plates were stained with 1% crystal violet and plaques were counted. The remaining culture supernatants were used to determine NO production from macrophages using Griess assay reagent system as described below. To determine repeatability, the experiment was performed two more times with similar results and the data were pooled.

#### Determination whether resiquimod treatment induces type 1 IFN activity in avian macrophages

To determine type 1 IFN activity following resiquimod treatment of macrophages, the type 1 IFN bioassay was performed as has been described previously [27, 28]. Briefly, the MQ-NCSU cells were cultured with LM-HAHN media in 12-well plates with 1 × 10^6^ cells per well for 24 h and stimulated with RPMI media containing resiquimod (10 μg/ml) or RPMI media only as control separately. Each group was treated in triplicates. The resultant MQ-NCSU cell culture supernatants were collected at 24 h post-treatment and 250 μl was transferred to DF-1 cell monolayers. Twenty-four hours later, the DF-1 cells were infected with VSV-GFP [27, 29] at a MOI of 0.1. At 24 h post infection, the cells were fixed with 4% paraformaldehyde for 20 min, washed with Hanks’ Balanced Salt Solution (HBSS) twice and stained with Hoechst 33342 (Image-iT™ LIVE Plasma Membrane and Nuclear Labeling Kit (I34406), Invitrogen, Eugene OR, USA). The percentage of cells expressing GFP was determined using Image-J software as described below. To determine repeatability, this experiment was performed two more times with similar results and the data were pooled.

#### Determination whether resiquimod induces IL-1β production in avian macrophages

MQ-NCSU cells were cultured on coverslips in 12-well plates with 1 × 10^6^ cells per well. After 24 h of culture, RPMI media containing resiquimod (10 μg/ml) or RPMI media only as a negative control were added separately. Each group was treated in triplicates. Protein transport inhibitor cocktail (2 μl/ml) (cocktail of Brefeldin A and Monensin, *eBioscience*, San Diego, CA, USA) was added to culture medium following 6 h of incubation in order to prevent release of IL-1β to the extracellular space. At 24 h, the cells were fixed with 4% paraformaldehyde and immunofluorescent staining was performed as described under a separate title below. The experiment was repeated two more times with similar results and the data were pooled.

#### Determination whether resiquimod-mediated inhibition of ILTV replication is attributable to IL-1β production from macrophages

MQ-NCSU cells were cultured in 12-well plates with 1 × 10^6^ cells per well for 24 h. The cells were incubated with RPMI media containing resiquimod (10 μg/ml) or RPMI media only (negative control). We used 6 replicates in each group. Cell culture supernatants were collected at 24 h post-treatment and 250 μl was transferred to LMH cell monolayers that were pre-incubated (30 min) with DMEM media containing 1.2 μg/ml IL-1 receptor antagonist (IL-1Ra) (Kingfisher Biotech, Inc., CITY MN, USA) (*n* = 3) or DMEM media only (without IL-1Ra) as a negative control (*n* = 3). The LMH cells were infected after 24 h with ILTV (300 PFU/well or MOI = 0.0012). The plates were stained with 1% crystal violet at 5 dpi and resulting plaques were counted. The experiment was repeated two more times with similar results and the data were pooled.

#### DNA extraction and real-time polymerase chain reaction (PCR)

DNA extraction was carried out from the swab samples collected at 4 and 7 dpi from the experiment performed to determine whether *in ovo* treatment of synthetic ssRNA elicits an antiviral response against ILTV post-hatch, using QIAamp DNA mini kit (QIAGEN GmbH, Hilden, Germany) as per manufacturer’s instructions. The extracted DNA was quantified using the Nanodrop 1000 spectrophotometer (ThermoScientific, Wilmington DE, USA) with absorbance at 260 nm wavelength.

For real-time PCR, 80 ng (cloacal swabs) or 25 ng (oropharyngeal swabs) of the extracted DNA was used to quantify the protein kinase (PK) gene (ORF2) of ILTV in relation to β actin housekeeping gene. The real-time PCR assay was conducted using a CFX96-C1000 Thermal Cycler (Bio-Rad Laboratories, Mississauga, ON, Canada) in a 96-well PCR plates (VWR, Edmonton AB, Canada). The cycling conditions were 95 °C for 20 s, followed by 40 cycles of 95 °C for 30 s and 60 °C for 30 s. Five pM of β actin primers (F: 5’-CAA CAC AGT GCT GTC TGG TGG TA-3′ and R: 5’-ATC GTA CTC CTG CTT GCT GAT CC -3′) [30, 31] and ILTV PK gene specific primers (F: 5’-TAC GAT GAA GCG TTC GAC TG -3′ and R: 5′-AGG CGT GAC AGT TCC AAA GT -3′) [32, 33] were used in a Fast SYBR® Green Master Mix (Invitrogen, Burlington ON, Canada) with a final reaction volume of 20 μL. As a positive control, the gene specific plasmids were included and as a negative control, DNAse/RNAse free water was included. Acquisition of fluorescent signals was performed at 60 °C for 30 s and the melting curve was analyzed at 95 °C for 10 s, 65 °C for 5 s and finally 95 °C for 5 s.

#### Determination of NO concentrations in culture supernatants and in lung homogenates

The NO concentrations in MQ-NCSU cell culture supernatants or in lung homogenates were assayed using Griess assay reagent system (Promega Corporation, Madison WI, USA) by measuring the end product, nitrite according to manufacturer’s instructions. Briefly, culture supernatants and Griess reagents (1% sulfanilamide and 0.1% N-1-naftyletylendiamin dihydrochloride) were mixed in equal volume in 96-well plates (in triplicate) and incubated for 10 min at room temperature. The mean absorbance value of each sample was determined using SpectraMax M2 microplate reader (Molecular devices, Sunnyvale, CA, USA) at the wavelength of 548 nm. Based on generated sodium nitrate standard curve along with the samples, the nitrite concentration of each sample was calculated.

#### Immunofluorescent assay

The indirect immunofluorescent assay was performed on the cells fixed on coverslips to determine IL-1β production from macrophages in vitro and in sectioned lung tissues (5 μm) originated from day 1 chicken preserved in OCT to quantify macrophage numbers. Slides were incubated for 30 min in block buffer: 5% goat serum in TBS buffer (Trizma base: 20 millimolar (mM), NaCl: 138 mM and distilled water, pH 7.6). Unlabeled mouse monoclonal antibody specific for chicken macrophages, KUL01 (Southern Biotech, Birmingham AL, USA) and unlabeled rabbit polyclonal antibody specific for chicken IL-1β (Bio-Rad Laboratories, Mississauga ON, Canada) were used in, respectively, 1:200 and 1:10 dilutions in block buffer and incubated for 30 min. For macrophage staining, DyLight® 550 conjugated goat anti-mouse IgG (H + L) (Bethyl Laboratories Inc., Montgomery TX, USA) was used in 1:500 dilution in block buffer as the secondary antibody and incubated for 1 h. For IL-1β staining, biotinylated goat anti-rabbit IgG(H + L) (Vectashield, Vector Laboratories Inc., Burlingame, CA, USA) was used in 1:400 dilution in block buffer and incubated for 1 h and subsequently incubated with DyLight® 488 in 1:67 dilution for 20 min. TBS-T buffer (TBS with 0.1% Tween 20) was used to wash the slides twice, followed by a single wash with PBS following each step. All the incubations were performed at the room temperature in a humidified chamber. Finally, the slides were mounted with coverslips using mounting medium containing 4′, 6-Diamidine-2′-phenylindole dihydrochloride (DAPI) (Vectashield, Vector Laboratories Inc., Burlingame, CA, USA) and sealed with lacquer.

The double immunofluorescent assay was performed in sectioned lung tissues (5 μm) preserved in OCT to determine IL-1β production from macrophages in vivo as described previously [34]. Briefly, the slides were incubated for 30 min in block buffer: 5% goat serum in TBS buffer (Trizma base: 20 millimolar (mM), NaCl: 138 mM and distilled water, pH 7.6). Unlabeled mouse monoclonal antibody specific for chicken macrophages, KUL01 (Southern Biotech, Birmingham AL, USA) was used 1:200 dilutions in block buffer and incubated for 30 min. For macrophage staining, DyLight® 550 conjugated goat anti-mouse IgG (H + L) (Bethyl Laboratories Inc., Montgomery TX, USA) was used in 1:500 dilution in block buffer as the secondary antibody and incubated for 1 h. Then for IL-1β staining, the slides underwent avidin-biotin blocking step (Vector Laboratories, Inc., Burlingame, CA, USA) of 15 min each before incubating for 30 min in block buffer. Then, unlabeled rabbit polyclonal antibody specific for chicken IL-1β (Bio-Rad Laboratories, Mississauga ON, Canada) were used in, 1:10 dilutions in block buffer and incubated for 30 min. As a secondary antibody for IL-1β, biotinylated goat anti-rabbit IgG(H + L) (Vectashield, Vector Laboratories Inc., Burlingame, CA, USA) was used in 1:400 dilution in block buffer and incubated for 20 min and subsequently incubated with DyLight® 488 for 20 min. TBS-T buffer (TBS with 0.1% Tween 20) was used to wash the slides twice, followed by a single wash with PBS following each step. All the incubations were performed at the room temperature in a humidified chamber. Finally, the slides were mounted with coverslips using mounting medium containing DAPI (Vectashield, Vector Laboratories Inc., Burlingame, CA, USA) and sealed with lacquer.

### Data analyses and statistics

To quantify the IL-1β production and numbers of macrophages in the examined samples, 5 areas with highest DyLight® 550 (macrophages) or DyLight® 488 (IL-1β) fluorescent signals and corresponding nuclear stained (DAPI) areas were captured under 40X magnification from each section. Then, these captured images were subjected to measure fluorescent signals using the Image-J software (National Institute of Health, Bethesda, Maryland, USA). For single macrophage staining and IL-1β staining, the resultant positive fluorescent signals for DyLight® 550 or DyLight® 488 as estimated by nuclear staining with DAPI were expressed relative to the total areas as a percentage. Analysis from double staining of macrophages and IL-1β was performed as described previously [34]. Briefly, the obtained images were subjected to measure fluorescent signals using the Image-J® software (National Institute of Health, Bethesda, Maryland, USA) and the resultant positive fluorescent signals for DyLight® 550 (macrophages), DyLight® 488 (IL-1β) signals, combined DyLight® 550 and DyLight® 488 signals (IL-1β positive macrophages) and the combined DyLight® 550 and DyLight® 488 signals with DAPI (total area of the section) were quantified. The combined macrophage and IL-1β signals were expressed relative to the total areas as a percentage.

For the purpose of identifying the differences between two groups, the Student’s *t*-test (GraphPad Prism Software 5, La Jolla, CA, USA) was used. The one-way analysis of variance (ANOVA) with Bonferroni’s post-test for selected comparison was performed to identify the differences between groups when more than two groups are parts of an experiment and the data is normally distributed. If the data is not normally distributed, Kruskal-Wallis non-parametric test with Dunn’s posttest for selected comparison was performed to identify the differences between groups. D’Agostino-Pearson omnibus normality test was performed to test normal distribution of the data. The outlier test was conducted before being analyzed with each set of data using the Grubbs’ test (GraphPad software Inc., La Jolla, CA, USA). The differences between groups were considered significant at *P* ≤ 0.05.

## Results

### Synthetic ssRNA, resiquimod recruits macrophages post-hatch in respiratory and gastrointestinal tracts when treated *in ovo*

**Fig. 1:**
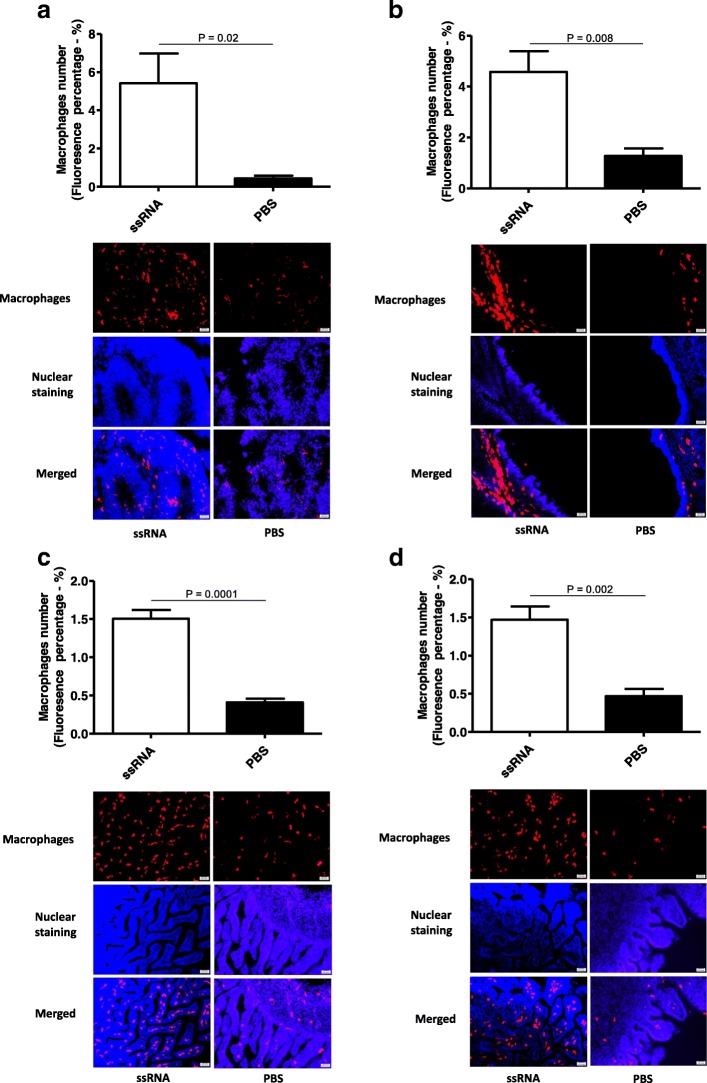
*In ovo* treated resiquimod increases macrophage recruitment in tissues of respiratory and gastrointestinal systems. SPF eggs were treated *in ovo* with resiquimod (*n* = 5) or PBS (*n* = 4) at ED18 and the eggs were incubated until hatch. At day 1 post-hatch, trachea, lungs, duodenum and large intestine were preserved in OCT, sectioned and immunofluorescent assay was performed with mouse monoclonal antibody specific for chicken macrophages, KUL01 (red signals) and with DAPI (blue signals) for nuclear staining. The quantitative data and representative immunofluorescent figures from each organ is shown, **a** lungs, **b** tracheas, **c** duodenum and **d** large intestine. Student’s *t-*test was performed to identify group differences and the differences were considered significant at *P* < 0.05

We examined trachea, lungs, duodenum and large intestine sections for macrophage recruitment following *in ovo* treatment of resiquimod or PBS. We found that *in ovo* treated resiquimod at ED18 increases macrophage numbers D1 post-hatch in lungs (*P* = 0.02), trachea (*P* = 0.008), duodenum (*P* = 0.0001) and large intestine (*P* = 0.002) when compared to PBS treated group (Fig. 1).

### *In ovo* treatment of resiquimod reduces ILTV shedding post-hatch

**Fig. 2:**
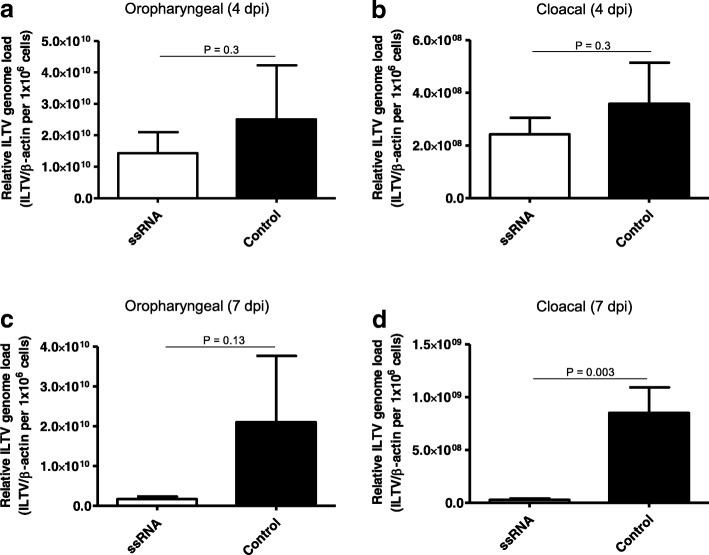
The effect of *in ovo* treated resiquimod on ILTV shedding post-hatch. **a** SPF eggs were *in ovo* treated with resiquimod (*n* = 6) or PBS (*n* = 6) at ED18, the eggs were incubated to hatch and infected with ILTV intra-tracheally at D1 post-hatch. Chicken oropharyngeal and cloacal swabs were collected and ILTV genome loads were determined. ILTV genome loads at 4 dpi **a** in oropharyngeal and **b** cloacal swabs, and at 7 dpi **c** in oropharyngeal and **d** cloacal swabs

We then investigated whether the observed macrophage response coincide with antiviral response against ILTV infection when encountered post-hatch. We found that resiquimod treated *in ovo* at ED18 shows a significant reduction in cloacal ILTV shedding at 7 dpi (Fig. 2d, *P* = 0.003) but not cloacal shedding at 4 dpi (Fig. 2b, *P* > 0.05) and oropharyngeal shedding at 4 and 7 dpi Fig. 2a and c, *P* > 0.05).

### Macrophages stimulated with resiquimod produces NO in vitro in a dose dependent way

**Fig. 3:**
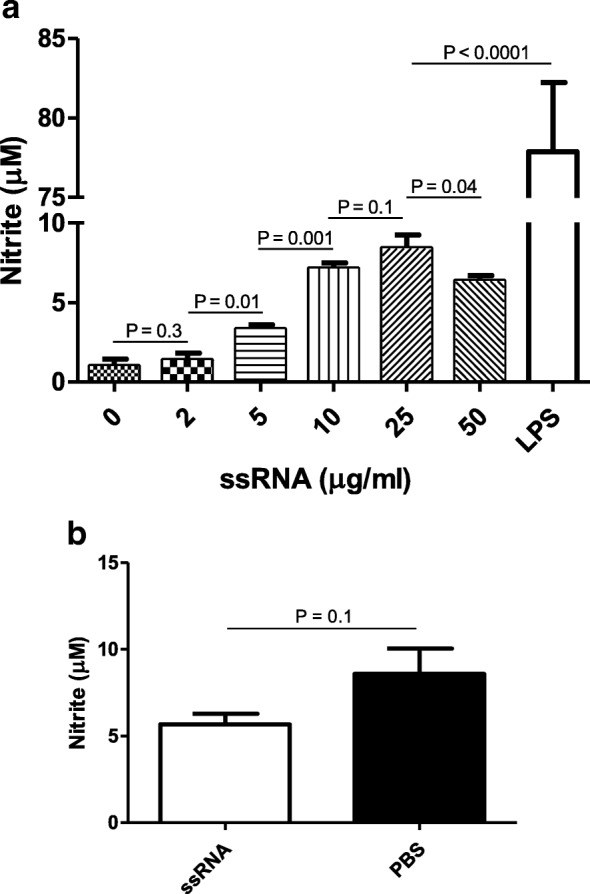
NO production from avian macrophages in vitro and from lungs in vivo following treatment with resiquimod. **a** The MQ-NCSU cells were cultured and treated with resiquimod along with a positive control (LPS) as indicated in the materials and methods. The doses of resiquimod are given on the horizontal axes and the determined nitrite concentrations are given on the vertical axes. Kruskal-Wallis test with Dunn’s post-test for selected comparison was performed to identify the differences between groups **b** SPF eggs were treated *in ovo* with resiquimod (*n* = 5) or PBS (*n* = 4) at ED18 and the eggs were incubated until hatch. At day 1 post-hatch, one lung was collected in PBS to perform NO assay. Student’s *t*-test was used to identify differences between two groups. The differences between groups were considered significant at *P* < 0.05

Since, previously it has been investigated the role of macrophages in vitro, specifically as NO producing cells during the response against avian viruses including ILTV [13, 14, 30, 35], we then evaluated the production of NO following resiquimod treatment of avian macrophages. We observed that stimulation of avian macrophages with resiquimod produced NO in a dose dependent manner (Fig. 3a).

### *In ovo* treatment of resiquimod did not lead to NO production in vivo

Since, we have observed that *in ovo* resiquimod treatment induces macrophages response in vivo and resiquimod stimulate avian macrophages to produce NO in vitro, we then evaluated to see whether *in ovo* administration of resiquimod induced NO production in vivo in lungs post-hatch. We found that resiquimod is not significantly inducing NO production in lungs in vivo (Fig. 3b, *P* > 0.05).

### Stimulation of avian macrophages with resiquimod inhibits ILTV replication independent of NO production

**Fig. 4:**
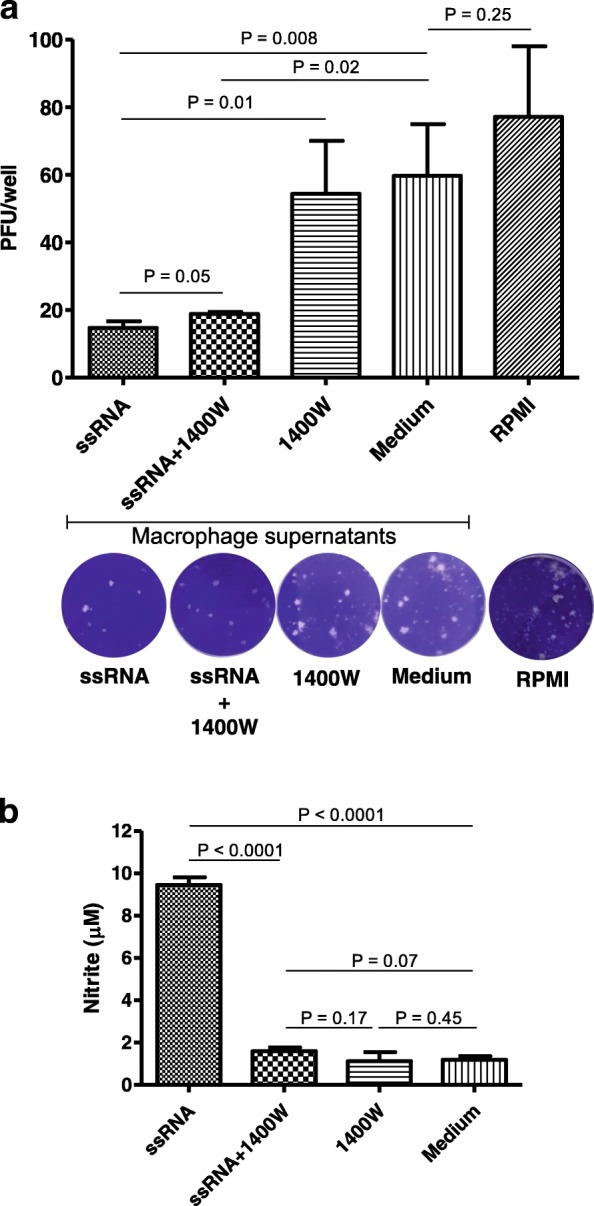
Antiviral effect of NO produced from avian macrophages following resiquimod treatment. **a** Avian macrophages, MQ-NCSU cells, were cultured in 12-well plates (1 × 10^6^ cells per well) for 24 h and stimulated with either resiquimod (10 μg/ml), resiquimod (10 μg/ml) plus 1400 W (100 μM), 1400 W (100 μM) or growth media (control) in triplicates. The culture supernatants were collected 24 h post-treatment, a portion was transferred to LMH cell monolayer grown in 12-well plates and infected with ILTV (300 PFU/well). **b** The NO assay was performed in remaining culture supernatants using Griess assay to quantify NO production. The experiment was repeated two more times with the same number of replicates with similar results and the data were pooled. Kruskal-Wallis test with Dunn’s post-test for selected comparison was performed to identify the differences between groups and the differences were considered significant at *P* < 0.05. **a** ILTV replication. **b** NO production

As a next step we investigated if stimulation of avian macrophages with resiquimod inhibits ILTV replication in vitro in a NO dependent way. Supernatants derived from macrophages following stimulation with resiquimod for 24 h were able to elicit antiviral response (Fig. 4a, *P* < 0.05) in LMH cells against ILTV which correlated with a significant increase in NO production from macrophages (Fig. 4b, *P* < 0.05) when compared to the untreated media controls. Furthermore, we observed that 1400 W-mediated inhibition of resiquimod-induced NO production in avian macrophages (Fig. 4b, *P* < 0.05) did not abrogate resiquimod-mediated antiviral response against ILTV (Fig. 4a, *P* > 0.05). Whereas, the increase in ILTV replication we observed in 1400 W group was higher than the viral replication observed in resiquimod groups (Fig. 4a, *P* < 0.05).

### Stimulation of avian macrophages with resiquimod increases the production of IL-1β but not type 1 IFNs

**Fig. 5:**
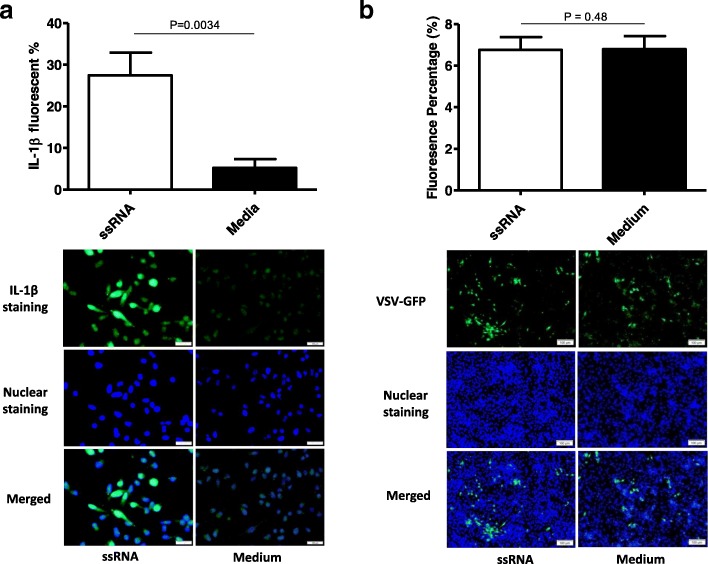
Stimulation of avian macrophages with the synthetic ssRNA, resiquimod increases the production of IL-1β but not type 1 IFNs. **a** Avian macrophages, MQ-NCSU cells, were cultured in 12-well plates (1 × 10^6^ cells per well) for 24 h and stimulated with either resiquimod (10 μg/ml) or growth media (control) in triplicates. The culture supernatants were collected 24 h post-treatment and transferred to DF-1 cell monolayers grown in 12-well plates and infected with VSV conjugated with GFP (0.1 MOI). At 24 h post infection, the percentage of cells expressing GFP signal was quantified after fixation with 4% paraformaldehyde and nuclear staining. The experiment was repeated two more times with similar results and the data were pooled. The representative figures in each group are shown. **b** The MQ-NCSU cells were cultured on coverslips in 12-well plates with 1 × 10^6^ viable cells per well and protein transport inhibitor (2 μl/ml) was added to culture medium after 6 h. After 24 h of culture, the cells were stimulated with resiquimod (10 μg/ml), or RPMI media as a control separately (triplicates per treatment). Following 24 h of treatment, immunofluorescent staining targeting IL-1β was performed after fixation with 4% paraformaldehyde. The experiment was repeated two more times with similar results and the data were pooled. The representative figures in each group are shown. Student’s *t*-test was used to identify differences between two groups and the differences were considered significant at *P* < 0.05

Next, we investigated to see whether other antiviral cytokines that are induced by resiquimod treatment of macrophages such as type 1 IFNs and IL-1β contribute to antiviral response against ILTV. We observed that the stimulation of macrophages with resiquimod had increased levels of intracellular IL-1β (Fig. 5a, *P* = 0.0034) in vitro but not type 1 IFN activity as measured by VSV-GFP type 1IFN assay (Fig. 5b, *P* = 0.48) in vitro when compared to untreated control group.

**Fig. 6:**
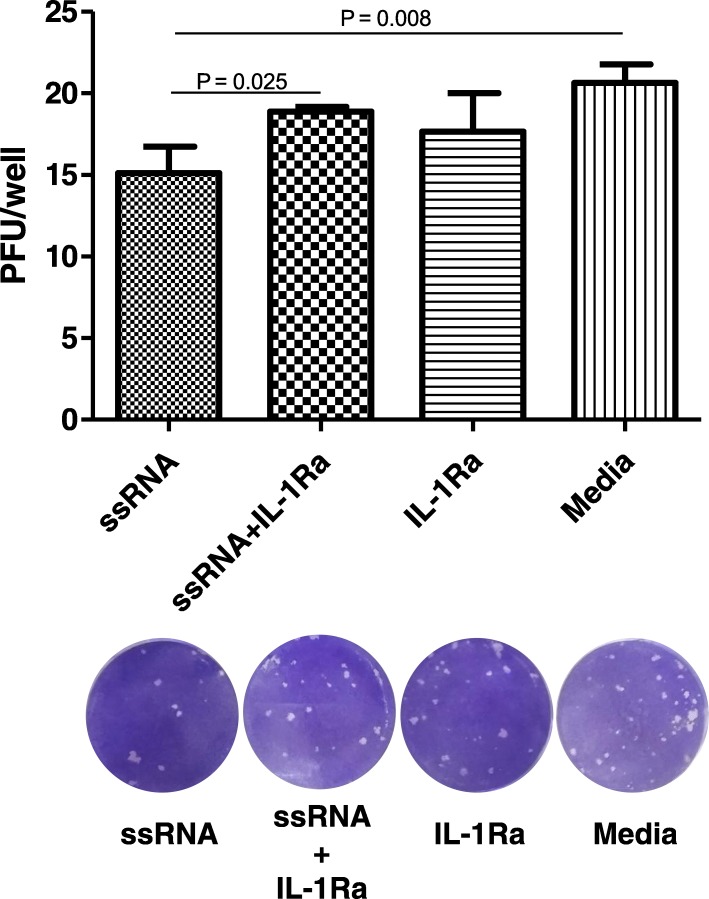
Antiviral activity of synthetic resiquimod against ILTV replication is not dependent on IL-1β production. Avian macrophages, MQ-NCSU cells, were cultured in 12-well plates (1 × 10^6^ cells per well) for 24 h and stimulated with either resiquimod (10 μg/ml) or growth media (control) in 6 replicates. The resultant MQ-NCSU cell culture supernatants were collected at 24 h post-treatment and transferred (250 μl) to LMH cell monolayer. Before transferring MQ-NCSU cell culture supernatants, 3 wells from resiquimod and control groups in LMH cells were incubated with 1.0 μg/ml of IL-1Ra for 30 min. Twenty-four hours later, the LMH cells were infected with ILTV (300 PFU/well). The plates were stained with 1% crystal violet at 5 dpi and resulting plaques were counted. The experiment was repeated two more times with the same number of replicates with similar results and the data were pooled. ANOVA with Bonferroni’s posttest for selected comparison was performed to identify the differences between groups and the differences were considered significant at *P* < 0.05

Next, we investigated to see whether resiquimod treatment *in ovo* can induce macrophages to produce IL-1β in vivo at day 1 post-hatch by double immunofluorescent staining for macrophages and IL-1β and found that *in ovo* resiquimod induces IL-1β production significantly from macrophages in lungs (Fig. 6a, *P* = 0.01).

### Antiviral response of resiquimod against ILTV replication is not attributable to IL-1β production

**Fig. 7:**
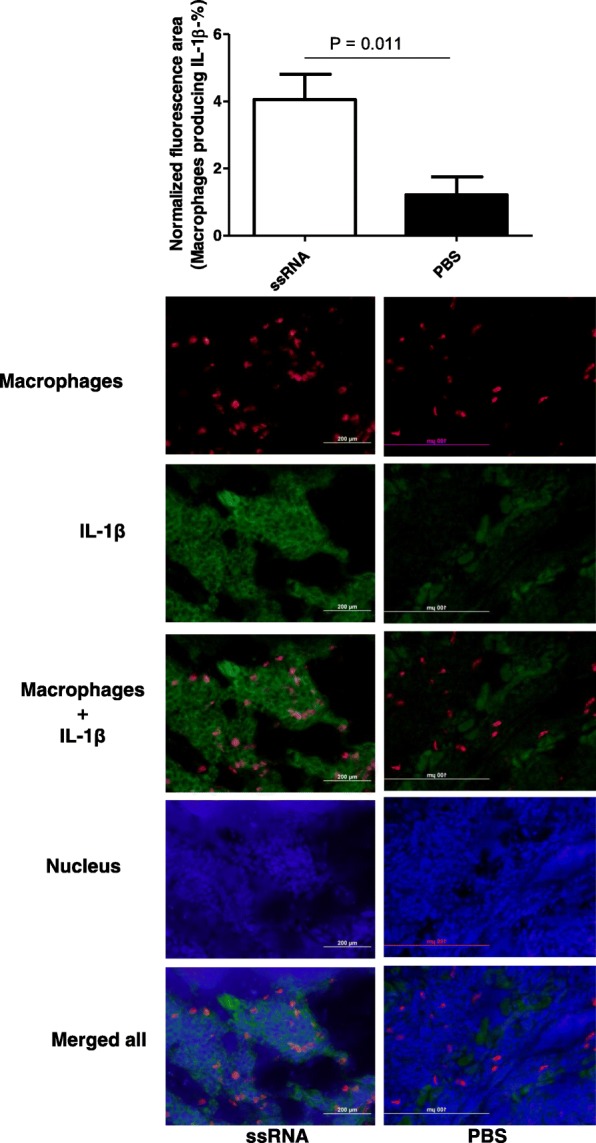
*In ovo* synthetic resiquimod treatment increases the production of IL-1β from macrophages in lungs in vivo*.* SPF eggs were treated *in ovo* with resiquimod (*n* = 5) or PBS (*n* = 4) at ED18 and the eggs were incubated until hatch. At day 1 post-hatch, lungs were preserved in OCT, sectioned and double immunofluorescent assay was performed to identify the macrophages that produce IL-1β. The quantitative data from lung is shown along with representative immunoassayed images. Student’s *t-*test was performed to identify group differences and the differences were considered significant at *P* < 0.05

We then investigated to see whether stimulation of avian macrophages with resiquimod inhibits ILTV replication in vitro in an IL-1β dependent manner. Here, we found that culture supernatants derived from macrophages following stimulation with resiquimod were able to inhibit ILTV replication compared to the media controls (Fig. 7, *P* < 0.05) and blocking IL-1β signaling using IL-1Ra not abrogated the antiviral response elicited against ILTV significantly (Fig. 7, *P* > 0.05). Furthermore, we observed that blocking the IL-1β response following resiquimod stimulation did not significantly increase the ILTV replication when compared to the group that received only media (Fig. 7, *P* > 0.05).

## Discussion

The findings of the study described here are three-fold. First, *in ovo* treated resiquimod can elicit macrophage response in respiratory and intestinal tracts of chicken when the eggs are hatched in addition to IL-1β production in lungs. However, *in ovo* treatment of embryonated eggs with resiquimod did not lead to decrease in ILTV shedding as demonstrated by oropharyngeal ILTV genome loads, although there was a significant decrease in cloacal ILTV shedding recorded only at 7 dpi. Second, resiquimod treatment of avian macrophages leads to antiviral response against ILTV in vitro. Third, we found that resiquimod induces NO and IL-1β production, but not type 1 IFN activity in vitro. The resiquimod-mediated antiviral response elicited against ILTV in avian macrophages is not dependent on any of the innate immune mediators observed.

Increased recruitment of macrophages has been observed previously in chickens following *in ovo* treatment of CpG DNA (TLR21 ligand) [13, 30] and LTA (TLR2 ligand) [15]. In the current study, we discovered that *in ovo* treatment of TLR7 ligand, synthetic ssRNA, increased recruitment of macrophages in respiratory and gastrointestinal systems. Macrophages are key immune cells involved in initiating innate antiviral response by several mechanisms. First, avian macrophages are capable of producing highly reactive antiviral molecules such as NO [36] and previously it has been reported NO mediated antiviral activity against number of avian viruses such as ILTV, reovirus and Marek’s disease virus [14, 35, 37]. Second, avian macrophages can produce a number of cytokines such as type 1 IFNs and IL-1β [38, 39]. These cytokines can elicit antiviral responses. For example, type I IFNs are effective in reducing avian viruses such as birna virus, corona virus, herpes viruses and paramyxovirus [40–44]. Third, macrophages play a role as phagocytic cells reducing virus burden in the host [45]. Fourth, as an antigen presenting cell, the macrophages play a vital role in activating the adaptive arm of the immune system by presenting antigens to helper T lymphocytes [46]. In the current study, we observed that avian macrophages are capable of producing NO and IL-1β following stimulation with resiquimod although further studies are required to elucidate whether resiquimod is involved in enhancing phagocytosis and antigen presentation increasing adaptive immune response against ILTV infection.

It has been reported previously that many TLR ligands were able to induce protective host responses against ILTV, such as CpG DNA, LPS and LTA [9, 14, 15, 30]. Although different types of TLR ligands were found to be effective against many viral infections, there is a paucity of literature on the antiviral effect of TLR7 ligand against avian viral infections. Previously, Annamalai et al reported that the TLR7 agonist, R848, induced protective antiviral response against infectious bursal disease virus infection in 3 weeks old chicken, associated with the upregulation of pro-inflammatory genes, such as IL-1β, IL-4, iNOS and IFNγ [18]. Similarly, Stewart et al demonstrated that the other TLR7 ligands, Poly-C and ioxoribine, inhibited low pathogenic H1N1 avian influenza virus replication in chickens and the antiviral response of ioxoribine was associated with the increased gene expression for IFNα, IFNβ and IFNλ from primary chicken splenocytes [47]. Our study was directed to demonstrate whether TLR7 ligand, resiquimod was able to elicit antiviral responses against ILTV infection when resiquimod treatment is done *in ovo.* In agreement with previous studies using other TLR ligands against ILTV infection [9, 14, 15, 30], we found that *in ovo* treated resiquimod is capable of eliciting antiviral response against ILTV infection in terms of reducing cloacal virus shedding at 7 dpi. Although we observed that resiquimod was effective when treated *in ovo* reducing ILTV shedding via cloacal route at 7 dpi, we did not observe a significant reduction in ILTV shedding at 4 dpi and via oropharyngeal route at 4 or 7 dpi. Previously, it has been reported in similar experiment setting that CpG DNA injected *in ovo* reduces ILTV cloacal shedding at 4 dpi but not the oropharyngeal shedding [48]. It is difficult to explain this discrepancy of ILTV shedding between cloacal and oropharyngeal routes. However, it may be potentially related to the differences in virus replication pattern between 4 dpi and 7 dpi, a differences in the antiviral mechanism induced from a different TLR ligand used [48] and the differences in the immune response between gastrointestinal and respiratory systems [49] such as intestinal immune tolerance [50] and presence of antiviral surfactant proteins in the lungs [51]. Furthermore, we did not observed severe clinical manifestations following ILTV (strain N-71851) infection in this experiment as has been seen previously by our group [30, 48]. The lack of clinical manifestation except transient non-specific signs such as huddling together and droopy and ruffled feathers may be due to differences in the batch of the virus and slight reduction in ILTV titer during storage at − 80 °C.

Furthermore, we observed that the culture supernatants originated from macrophages stimulated with resiquimod in vitro is capable of inhibiting ILTV replication. Although resiquimod was capable of inducing NO production in vitro, interestingly, we did not find the resiquimod-mediated induction of NO in vivo*.* Furthermore, the resiquimod-mediated NO production is not eliciting antiviral response against ILTV. However, previously it has been shown that NO originated from LPS/ CpG DNA-mediated induction of avian macrophages is inhibitory against ILTV and low pathogenic avian influenza virus infections [13, 14]. Although, this discrepancy in antiviral response mediated by NO originated from various TLR pathways is difficult to explain, it is possible that the difference may be connected to the amount of NO production downstream of these TLR pathways. In the current study, the amount of NO produced following resiquimod induced macrophages was minimal (< 10 μM) compared to other TLR ligands we tested previously in similar experimental setting such as LPS (> 50 μM) [14] and CpG DNA (> 30 μM) [13]. Overall, our current and previous findings suggest that antiviral response mediated by NO is dependent on the concentration of NO. However, one of the limitation in this study is that we did not find the exact level of NO that can decrease the viral replication and that 1400 W can inhibit this antiviral effect by including other TLR ligands such as LPS and CpG DNA at different concentrations.

Type 1 IFNs are important antiviral cytokines produced downstream of TLR signaling involved in antiviral host response that has been demonstrated in a number of host-viral infection models [52, 53]. In mice, it has been reported that the activation of TLR7 increased production of type 1 IFNs (IFNα) [54]. In chickens, type 1 IFNs produced following TLR3 ligand, dsRNA has been shown to inhibit avian viruses such as avian paramyxovirus, avian influenza virus and Marek’s disease virus [55–57]. However, we found that no significant induction of type 1 IFNs production following stimulation of avian macrophages with resiquimod. It is difficult to explain this discrepancy between different species and between different TLR ligands. However, it may be potentially related to the differences in the pathways that are activated, which requires further investigation.

Previously, it has been shown that avian macrophages are a source of IL-1β mRNA following stimulation with TLR21 ligand, CpG DNA [30]. Our current data show that macrophages produce IL-1β in response to TLR7 ligand, resiquimod in vitro and in vivo in lungs. Direct and indirect IL-1β mediated antiviral responses have been observed in other host-virus infection models. Furthermore, it has been reported that IL-1β inhibits the replication of West Nile virus [58], hepatitis B virus [59], cytomegalovirus [60] and respiratory syncytial virus [61]. However, our current data show that the antiviral response against ILTV infection is not dependent on IL-1β originated from avian macrophages in response to resiquimod. Whether this lack of antiviral responses against ILTV attributable to IL-1β is due to the amount of IL-1β produced following resiquimod treatment requires further investigation. This fact would have been clarified in our in vitro experiment if we had included recombinant IL-1β as a positive control. Although, our finding of resiquimod-induced antiviral response against ILTV is not dependent on individual innate immune mediators, it may be potentially due to the combined effect of NO and IL-1β or other antiviral cytokines not tested in this study such as IFNγ [62].

This study describes that the *in ovo* administration of resiquimod stimulates the innate immune system as indicated by the expansion of macrophage numbers and increased production of innate antiviral molecules such as NO and IL-1β. Consequently, resiquimod treatment resulted in the reduced ILTV shedding via cloacal route that can minimize transmission potential of the disease*.* The main implication of this observation is that this induction of immune response via *in ovo* administration is desirable in the field situations in order to provide early innate immune response at the time of placing the day-old chickens in the barn against circulating environmental pathogens. Although we studied the mechanistic aspects of *in ovo* resiquimod treatment against only ILTV infection, our finding of the mechanisms of induction of innate immunity following *in ovo* treatment of resiquimod may be applicable against other respiratory viruses as well due to the non-specific nature of innate host responses which requires further investigation.

## Conclusions

In conclusion, we have shown that the administration of TLR7 ligand, resiquimod prophylactically *in ovo* reduces ILTV cloacal shedding (at 7 dpi) correlating with increased macrophage recruitment and IL-1β expression. In vitro, we found that although enhanced NO and IL-1β productions from avian macrophages are possible following resiquimod stimulation, antiviral response against ILTV is not dependent on individual mediators observed. It is possible that the cells other than macrophages are involved in vivo in eliciting IL-1β expression. Although our results provide insights into the mechanisms of antiviral response mediated by resiquimod against ILTV infection in chickens, further investigations are required to identify further cells and mediators involved in enhanced antiviral response against ILTV infection following administration of resiquimod *in ovo*.

## Abbreviations

1400 W: N-([3-(Aminomethyl) phenyl] methyl) ethanimidamide dihydrochloride
DAPI: 6-Diamidine-2′-phenylindole dihydrochloride
DF: Douglas Foster
DMEM: Dulbecco’s Modified Eagle’s Medium
dpi: Days post-infection
ED: Embryo day
FBS: Fetal bovine serum
GFP: Green fluorescent proteins
HBSS: Hanks’ Balanced Salt Solution
IFN: Interferon
IL: Interleukin
IL-1Ra: IL-1 receptor antagonist
ILTV: Infectious laryngotracheitis virus
iNOS: Inducible nitric oxide synthase
LMH: Leghorn chicken hepatocellular carcinoma
LPS: Lipopolysaccharide
LTA: Lipotechoic acid
MOI: Multiplicity of infection
MQ-NCSU: Muquarrab Qureshi-North Carolina State University
NO: Nitric oxide
OCT: Optimum cutting temperature
PAMP: Pathogen-associated molecular patterns
PBS: Phosphate buffered saline
PCR: Polymerase chain reaction
PFU: Plaque forming units
PK: Protein kinase
PRR: Pattern recognition receptors
RPMI: Roswell Park Memorial Institute
SPF: Specific pathogen free
ssRNA: Single stranded ribonucleic acid
TLR: Toll-like receptor
VSV: Vesicular stomatitis virus
